# Strategies for reducing surgical site infection in neurosurgery: experience from a Brazilian hospital

**DOI:** 10.1186/2047-2994-4-S1-P79

**Published:** 2015-06-16

**Authors:** R Saad, P Vidal, S Lessa, V Bastos, R Ito, C Genari, C Vasconcelos, M Queiroz

**Affiliations:** 1 Infectious Diseases, Hospital São Camilo, São Paulo, Brazil; 2 ICU, Hospital São Camilo, São Paulo, Brazil; 3 Anesthesiology, Hospital São Camilo, São Paulo, Brazil

## Introduction

Our Hospital focus on providing excellent care for surgical patients, with approximately 9000 surgeries happening in our Operating Rooms (OR) every year.

## Objectives

To describe the strategies adopted to reduce Surgical-Site infection (SSI) in patients undergoing neurosurgery.

## Methods

In April, 2013, we observed a peak of SSI in our neurosurgical patients (21.4%). Looking back on our data, we noticed that SSI were higher in this population when compared to our general clean Surgery population (9.6% X 1.4%) in the first semester of 2013, and we needed to understand the OR practices to identify and to implement possible points for improvement. After an evaluation of all the process, our main actions consisted on: improving skin preparation in Neurosurgical patients; reducing time of pre-operative preparation; review antibiotic prophylaxis guidelines (drug of choice/time to start/time to do additional doses/length of ABT prophylaxis); discussion of individual cases of infection with the surgical team.

## Results

In the first semester of 2013 we did extensive on-site work with the Neurosurgeons at our Hospital as well as with the OR staff. In the months that followed, we observed a reduction in the number of SSI infections in the Neurosurgical patients (6.7% in the second semester of 2013 and 2,3% in the first semester of 2014) accompanied with a reduction in the SSI in global surgery infection (1.31% in 2013 and 0.5% in the first semester of 2014) as it would be expected.

## Conclusion

With different actions in important points of the care process for patients undergoing neurosurgery, we managed to get success with a significant decrease in infections in this group of patients. We highlight the involvement of neurosurgeons in improving the process.

## Disclosure of interest

None declared.

